# Supplementary material for: HIVprotI: an integrated web based platform for prediction and design of HIV proteins inhibitors
Source: J Cheminform. 2018 Mar 9;10:12. doi: 10.1186/s13321-018-0266-y (PMC5845081; doi:10.1186/s13321-018-0266-y)
Supplement: Supplementary file 2 — Additional file 2. Source code of HIVProtI web server. [file 13321_2018_266_MOESM2_ESM.zip › HIVprotI_Source-code/hivprotI/weka-3-6-6/doc/weka/core/PropertyPath.html]

PropertyPath


---


|  |  |  |
| --- | --- | --- |
| **Overview** \| **Package** \| **Class** \| **Tree** \| **Deprecated** \| **Index** \| **Help** \| **Weka's home** | |  |
| **PREV CLASS**   **NEXT CLASS** | **FRAMES**    **NO FRAMES**     **All Classes** |  |
| SUMMARY: NESTED \| FIELD \| CONSTR \| METHOD | DETAIL: FIELD \| CONSTR \| METHOD |  |


---


## weka.core Class PropertyPath

```
java.lang.Object
  weka.core.PropertyPath
```

**All Implemented Interfaces:**: RevisionHandler

---

``` public class PropertyPath extends java.lang.Object implements RevisionHandler ```

A helper class for accessing properties in nested objects, e.g., accessing
the "getRidge" method of a LinearRegression classifier part of
MultipleClassifierCombiner, e.g., Vote. For doing so, one needs to
supply the object to work on and a property path. The property path is a
dot delimited path of property names ("getFoo()" and "setFoo(int)" have
"foo" as property name), indices of arrays are 0-based. E.g.:

`getPropertyDescriptor(vote, "classifiers[1].ridge")` will return
the second classifier (which should be our LinearRegression) of the given
Vote meta-classifier and there the property descriptor of the "ridge"
property. `getValue(...)` will return the actual value of the
ridge parameter and `setValue(...)` will set it.

**Version:**
:   $Revision: 4742 $

**Author:**
:   fracpete (fracpete at waikato dot ac dot nz)

---

| **Nested Class Summary** | |
| --- | --- |
| `static class` | `PropertyPath.Path`             Contains a (property) path structure |
| `static class` | `PropertyPath.PathElement`             Represents a single element of a property path |


**Constructor Summary**

`PropertyPath()`


| **Method Summary** | |
| --- | --- |
| `static weka.core.PropertyPath.PropertyContainer` | `find(java.lang.Object src, PropertyPath.Path path)`             returns the property and object associated with the given path, null if a problem occurred. |
| `static java.beans.PropertyDescriptor` | `getPropertyDescriptor(java.lang.Object src, PropertyPath.Path path)`             returns the property associated with the given path, null if a problem occurred. |
| `static java.beans.PropertyDescriptor` | `getPropertyDescriptor(java.lang.Object src, java.lang.String path)`             returns the property associated with the given path |
| `java.lang.String` | `getRevision()`             Returns the revision string. |
| `static java.lang.Object` | `getValue(java.lang.Object src, PropertyPath.Path path)`             returns the value specified by the given path from the object |
| `static java.lang.Object` | `getValue(java.lang.Object src, java.lang.String path)`             returns the value specified by the given path from the object |
| `static void` | `main(java.lang.String[] args)`             for testing only |
| `static boolean` | `setValue(java.lang.Object src, PropertyPath.Path path, java.lang.Object value)`             set the given value specified by the given path in the object |
| `static void` | `setValue(java.lang.Object src, java.lang.String path, java.lang.Object value)`             set the given value specified by the given path in the object |

**Methods inherited from class java.lang.Object**

`equals, getClass, hashCode, notify, notifyAll, toString, wait, wait, wait`

**Constructor Detail**

### PropertyPath

```
public PropertyPath()
```


**Method Detail**

### find

```
public static weka.core.PropertyPath.PropertyContainer find(java.lang.Object src,
                                                            PropertyPath.Path path)
```

:   returns the property and object associated with the given path, null if
    a problem occurred.

    :   **Parameters:**: `src` - the object to start from: `path` - the path to follow **Returns:**: not null, if the property could be found

---


### getPropertyDescriptor

```
public static java.beans.PropertyDescriptor getPropertyDescriptor(java.lang.Object src,
                                                                  PropertyPath.Path path)
```

:   returns the property associated with the given path, null if a problem
    occurred.

    :   **Parameters:**: `src` - the object to start from: `path` - the path to follow **Returns:**: not null, if the property could be found

---


### getPropertyDescriptor

```
public static java.beans.PropertyDescriptor getPropertyDescriptor(java.lang.Object src,
                                                                  java.lang.String path)
```

:   returns the property associated with the given path

    :   **Parameters:**: `src` - the object to start from: `path` - the path to follow **Returns:**: not null, if the property could be found

---


### getValue

```
public static java.lang.Object getValue(java.lang.Object src,
                                        PropertyPath.Path path)
```

:   returns the value specified by the given path from the object

    :   **Parameters:**: `src` - the object to work on: `path` - the retrieval path **Returns:**: the value, null if an error occurred

---


### getValue

```
public static java.lang.Object getValue(java.lang.Object src,
                                        java.lang.String path)
```

:   returns the value specified by the given path from the object

    :   **Parameters:**: `src` - the object to work on: `path` - the retrieval path **Returns:**: the value, null if an error occurred

---


### setValue

```
public static boolean setValue(java.lang.Object src,
                               PropertyPath.Path path,
                               java.lang.Object value)
```

:   set the given value specified by the given path in the object

    :   **Parameters:**: `src` - the object to work on: `path` - the retrieval path: `value` - the value to set **Returns:**: true if the value could be set

---


### setValue

```
public static void setValue(java.lang.Object src,
                            java.lang.String path,
                            java.lang.Object value)
```

:   set the given value specified by the given path in the object

    :   **Parameters:**: `src` - the object to work on: `path` - the retrieval path: `value` - the value to set

---


### getRevision

```
public java.lang.String getRevision()
```

:   Returns the revision string.

    :   **Specified by:**: `getRevision` in interface `RevisionHandler`
    :   **Returns:**: the revision

---


### main

```
public static void main(java.lang.String[] args)
                 throws java.lang.Exception
```

:   for testing only

    :   **Parameters:**: `args` - the commandline options - ignored **Throws:**: `java.lang.Exception` - if something goes wrong


---


|  |  |  |
| --- | --- | --- |
| **Overview** \| **Package** \| **Class** \| **Tree** \| **Deprecated** \| **Index** \| **Help** \| **Weka's home** | |  |
| **PREV CLASS**   **NEXT CLASS** | **FRAMES**    **NO FRAMES**     **All Classes** |  |
| SUMMARY: NESTED \| FIELD \| CONSTR \| METHOD | DETAIL: FIELD \| CONSTR \| METHOD |  |


---
